# Chronic Pain following Chest Trauma: Prevalence, Associated Factors, and Psychosocial Impact

**DOI:** 10.1155/2020/1030463

**Published:** 2020-01-28

**Authors:** Mohamed Kahloul, Imene Kacem, Mohamed Mehdi Sboui, Olfa El Maalel, Hana Daami, Meriam Hafsia, Manel Limam, Sana Aissa, Imene Ben Kbaier, Nejib Mrizak, Ajmi Chaouch, Walid Naija

**Affiliations:** ^1^Department of Anesthesia and Intensive Care, Sahloul Teaching Hospital, Sousse, Tunisia; ^2^Université de Sousse, Faculty of Medicine “Ibn El Jazzar”, Sousse, Tunisia; ^3^Department of Occupational Medicine, Farhat Hachad Teaching Hospital, Sousse, Tunisia; ^4^Department of Family and Community Medicine, Research Laboratory “LR12ES03”, Faculty of Medicine of Sousse, Sousse, Tunisia; ^5^Departement of Pneumology, Farhat Hached Teaching Hospital, Sousse, Tunisia

## Abstract

**Background:**

Chronic pain (CP) is a real public health concern. It is a common cause of poor quality of life and workplace absenteeism. It is well studied in many medical and surgical fields. However, only few data are available as regards to its occurrence in trauma patients.

**Purpose:**

To assess the prevalence, associated factors, and psychosocial impact of CP following chest trauma.

**Methods:**

This is an observational, descriptive, and analytic cross-sectional study performed in a Tunisian department of anesthesia and intensive care over a two-month period. Adult patients admitted one year ago for isolated chest trauma were enrolled. Data were collected by a phone interview. Studied variables were sociodemographic characteristics, traumatic injuries and their management, the occurrence of CP, and its psychosocial impact. CP was diagnosed by the Brief Pain Inventory (BPI) considering an evolution period of at least 3 months. Its impact was assessed by the BPI and the Posttraumatic stress disorder Checklist Scale (PCLS).

**Results:**

Fifty-four patients were included in the study. The prevalence of CP was 79.6%. The average CP intensity was 3.18 ± 1.4. It was neuropathic in 90.7%. Its main associated factors were pleural effusion (*p*=0.016), time to ICU admission (*p*=0.016), time to ICU admission (*p*=0.016), time to ICU admission (*p*=0.016), time to ICU admission (

**Conclusion:**

CP following chest trauma is frequent and severe requiring preventive measures such as high risk patients screening, better management of acute pain, and a multidisciplinary approach for patients with diagnosed CP.

## 1. Introduction

Chronic pain is defined as “pain that persists or recurs for more than 3 months” [1]. It is a real public health problem because of its high prevalence and its significant socioeconomic impact. It is a common cause of human suffering, poor quality of life, and workplace absenteeism [2, 3]. About 30% of adults in developed countries report persistent pain that causes chronic discomfort, significant functional limitations, and abusive or inappropriate drug use [2, 3].

This painful experience is well recognized in many medical fields. However, it is still an emerging problem in surgical and trauma contexts despite its effects on postoperative satisfaction and injured patients prognosis [4–7]. In addition, it seems to have a higher incidence after severe acute painful injuries such as chest trauma which includes any form of physical injury to the chest. Its reported incidence is about 10% of trauma admissions. It is the third most common cause of traumatic death, after head and spinal cord injury, with a mortality rate ranging from 10% to 60%. Typically, chest injuries are caused by blunt or penetrating mechanisms. Rib fractures are the most common lesions as they are diagnosed in about 50% of patients hospitalized after chest trauma. They commonly result in severe and prolonged painful experience that requires preventive strategies especially for high risk patients and effective therapeutic measures guided by a targeted screening approach [8].

Thus, this study was conducted to assess the prevalence, associated factors, and psychosocial impact of chronic pain following chest trauma.

## 2. Methods

After the ethics committee approval and the oral patients' consent, this observational, descriptive, and analytical study was performed in a Tunisian department of anesthesia and intensive care over a two-month period (from 15/02/2018 to 15/04/2018).

All survived patients admitted from 01/10/2016 to 30/09/2017 for isolated chest trauma were enrolled in the study. Their identification was based on the department admissions registers. Exclusion criteria were age below 18 years, history of CP prior to chest trauma, psychiatric disorders, cognitive impairments, communication difficulties, and patients who had have surgery or other traumatic injuries after being discharged from our department. Patients who could not be contacted by phone despite three attempts were also excluded.

Data were collected using a pre-established questionnaire considering both medical records and telephone interviews. Studied variables included patients' sociodemographic characteristics; the diagnosis and management of chest trauma; patients' outcome; the occurrence of CP and its psychosocial impact.

The diagnosis and psychosocial impact of CP were based on the Brief Pain Inventory (BPI) in its French version [9, 10]. This questionnaire contains 8 items exploring the main characteristics of CP (location, intensity, prescribed analgesic treatment, and evolution under treatment) and one item exploring its psychosocial impact on general activity, mood, walking ability, normal work, relations with others, sleep, and enjoyment of life. Chronicity was defined by a threshold of 3 months. The neuropathic nature of CP was assessed by a validated Arabic version of the DN4 questionnaire [11, 12].

Posttraumatic Stress Disorder (PTSD) screening was performed using the Posttraumatic stress disorder Checklist Scale (PCLS) in its validated French version [13]. This is a 17-item self-report measure reflecting DSM-IV symptoms of PTSD [14]. These items can be grouped into 3 subscales exploring intrusion (items 1 to 5), avoidance (items 6 to 12), and hyperstimulation (items 13 at 17).

The variables were analyzed using SPSS 20.0 software. Qualitative variables were expressed in numbers and percentages. Quantitative variables were presented either as mean ± standard deviations or as medians and range of extreme values according to their distribution. The comparison of means was done using Student's *t*-test for two independent series means and Snedecor's F-test of parametric variance analysis (one-way ANOVA) for the comparison of several means. The comparison of frequencies was done using the Pearson chi-square test.

The analyze of the relationship between two quantitative variables was performed using the Pearson correlation coefficient.

For the multivariate study, we used a multiple linear regression when the dependent variable is quantitative and a binary logistic regression when the dependent variable is qualitative. The inclusion of independent variables in the regression models was performed when their degree of significance was less than 0.2. For all statistical tests, the significance level *p* was set at 0.05.

## 3. Results

**Figure 1:**
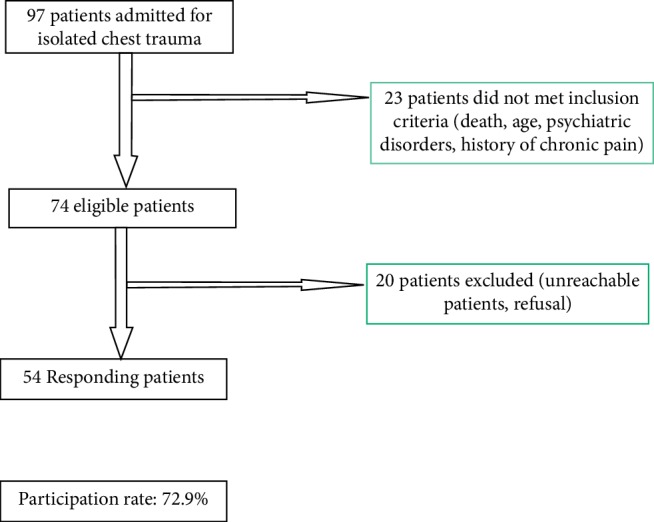
Chart flow.

Fifty-four patients were enrolled in this study corresponding to a participation rate of 72.97% (Figure 1). The mean age was 50.3 ± 18.65 years. The sex ratio was 5.75. The most common trauma circumstance was road accidents (53.7%). The most common injury type was rib fractures (92.6% of cases). The others sociodemographic and traumatic characteristics are summarized in Table 1.

The mean time to intensive care unit admission was 1.27 ± 1.03 days. Systemic analgesia was prescribed for all patients (paracetamol alone in 2 cases, paracetamol-nefopam in 16 cases, and paracetamol-nefopam-morphine in 36 cases). Only two patients had thoracic epidural analgesia. Oxygen therapy has been prescribed for all patients. Noninvasive ventilation was prescribed for 20 patients (38.8%). None of the patients required mechanical ventilation.

Pleural drainage was performed in 6 out of 38 patients with pleural effusions. The mean duration of drainage was 6.8 ± 2.8 days. Only one patient required thoracic surgery for recurrent pneumothorax. The mean duration of ICU hospitalization was 6.6 ± 3.8 days.

Considering the mean of the four pain intensity assessments of the BPI questionnaire, CP following chest trauma was diagnosed in 43 patients (79.6%). An overall mean score of CP intensity, evaluated by the simple numerical scale, was 3.18 ± 1.4. The characteristics of this pain are summarized in Table 2.

Associated factors with CP according to the univariate analysis were pleural effusion (89.37% vs 56.3%, *p*=0.016), delay in ICU admission less than 24 hours (83.7% vs 40%, *p*=0.05), and the occurrence of PTSD (100% vs. 68.6%, *p*=0.017) (Table 3). The pain was more frequent in the case of a fluid component effusion (pneumothorax 16/19 or 84.2%, hemothorax 14/15 or 93.3%, and hemo-pneumothorax 4/4 or 100% of cases). CP was less frequent in patients who had epidural analgesia (50% vs. 91.7%) without statistically significant difference (*p*=0.36).

After a multivaried analysis, only pleural effusion was independently associated factors with CP (*p*=0.01; OR = 6.9 CI 95% [1.2–37.3]).

Among the 43 patients with CP, the mean PCLS score was 36.58 ± 10. Probable or very likely PTSDs were noted, respectively, in 10 and 9 cases. The mean scores of the subdimensions were 13.67 ± 5.78 for intrusion, 15.48 ± 5.86 for avoidance, and 7.41 ± 2.96 for hyperstimulation.

Regarding the psychosocial impact of CP, the most commonly affected dimensions were general activity, work, sleep, and mood. The majority of patients rated this impact as moderate (≤3) (Table 4).

## 4. Discussion

CP following trauma is an emerging concept. Its frequency and psychosocial impact can worsen the prognosis of trauma, which is already a major public health problem. Preventive strategies should focus in its main risk factors. That is why this study could be considered as a part of quality processes looking for the promotion of trauma patients' care. However, it has some limitations that must be considered before result interpretation.

In fact, the sample size was relatively small but without real impact on the fiability of statistical analysis. However, it could be improved by a multicentric approach and a better sensitization of patients to participate in scientific investigations. Similarly, our results cannot be generalized because of selection bias since only patients hospitalized in intensive care unit were included.

In addition, the phone interview cannot replace a face-to-face approach. However, this investigative procedure has been considered by several studies [15–18] as it does not interfere with a good semiological description of patients functional complaints [19] and as it offers many facilities to participants. Other alternatives have been described in the literature such as postal or electronic mails at the expense of a selection or measurement bias since they require a certain intellectual level [20–22].

Finally, several scales were used in this study without having been previously validated on Tunisian populations. In fact, it is widely established that the performance of a score can deteriorate when it is used in a population other than the one at the origin of its creation. However, in a study validating the psychometric characteristics of BPI, Serlin et al. [23] did not find any bias related to linguistic or cultural differences. For this same scale, two versions are available including a long version evaluating the pain characteristics over a one- week-period, adapted for intrahospital use and a short version evaluating variables in only 24 hours, adapted for ambulatory use. This abridged version was validated in a large American study conducted in 2014 in several centers to evaluate noncancerous CP and its psychoemotional and social impacts in outpatients [24].

Moreover, compared with several other validated tools such as the Saint-Antoine questionnaire and the McGill Pain questionnaire [25, 26], the BPI seems to be simpler, easier, and more practical.

As for the DN4 questionnaire developed by Bouhassira [27] to diagnose neuropathic pain, an Arabic validated version is already available. For the PCLS, we used a French version developed and validated by Paul et al. [28].

Considering these scales, the diagnosis of CP beyond the third month post-chest trauma was established in 43 patients with a prevalence of 79.7%. This finding is comparable to several published results.

According to an American study enrolling 101 trauma patients with unspecified injuries types, the prevalence of CP assessed 4 months after the trauma using the BPI-SF scale was 79.2% [29].

It was 60% in the retrospective cohort of Rabiou et al. [30] including 56 patients admitted to a Moroccan military hospital for blunt chest trauma with at least one rib fracture.

Similar results were found by Fabricant et al. in their prospective study analyzing 203 patients hospitalized for chest trauma [31]. CP was assessed by the MC Gill questionnaire (MPQ), at 2 months of trauma, in 187 patients. It was retained in 110 patients, corresponding to a prevalence of 59%.

However, much lower prevalence has also been reported in the literature. According to Gordy et al. [32], the prevalence of CP assessed by the McGill Pain Questionnaire at 6 months of trauma was 22%. It was 22.5% in a Singapore study enrolling 102 patients with rib fractures [33].

In a Canadian study conducted in 57 trauma centers over a 10-year period, the CP assessed at the third posttraumatic month in 95134 patients had a prevalence of 15.3% [7].

On the other hand, higher results have also been reported. In a literature review conducted in 2009, analyzing the series of polytrauma patients with associated spine injury, the prevalence of post-traumatic CP varied from 26% to 96% [34].

This great variability found for prevalence related results, has also been reported for CP semiological characteristics such as intensity and type.

In our study, the mean score of the lowest CP intensity was 1.9 while it was 4.7 for the most severe CP. The mean pain intensity in general was 3.18 ± 1.4. Gordy et al. [33] found a mean CP score of 3. However, Kerr-Valentic et al. [35] reported a mean CP score equal to 1. In the study by Rabiou et al. [30], the pain intensity was low (VRS < 3) in 23% of cases, moderate (VRS between 3 and 7) in 37% of cases, and severe (VRS > 7) in 40% of cases.

As for the CP type, data of the literature are quite rare and contradictory. It was neuropathic in 90.7% of our patients. However, in the study of Rabiou et al. [30], the neuropathic character was reported only in 6.25% of cases.

The great disparity of results in the literature reflects the complexity of the concept of CP in traumatology particularly the thoracic one. Indeed, the definitions considered to designate CP have not been consensual at least for the duration required to retain chronicity. This duration varied from 3 months in the study of Rabiou et al. [30] and Daoust et al. [7] to 4 months in the study of Trevino et al. [36] to 6 months in the study of Shelat et al. [32] and up to 1 year in the study of Gordy et al. [33].

In addition, the prevalence and characteristics of CP may vary with the severity of anatomical injuries and the extent of tissue damage. Thus, the prevalence decreased from more than 79% in the study of Trevino et al. [29] including violent chest trauma to less than 16% in the study of Daoust et al. [7] including less severe chest trauma. Finally, some methodological variability should be considered such as the tool used in pain assessment, the approach considered in data collection and the characteristics of the studied population.

These explanations could also reveal the variability of risk factors reported in the literature. In our study, only pleural effusion was significantly associated with CP (*p*=0.024).

The effect of anatomical injury types was analyzed by Daoust et al. [7], who found a higher CP risk with spinal cord injuries (OR = 3.94) and discovertebral trauma (OR 1.58). These findings support the effect of nerve damage in the genesis of CP as it has been advanced by several researchers such as Moseley and Butler [37] and Wallwork et al. [38].

However, the number of fractured ribs, their location, and degree of stability as well as the presence of effusion or the severity of the trauma have not been identified as risk factors in several others studies [30, 33]. This discrepancy should be analyzed considering the severity of the anatomical damage which was assessed by validated tools in very few publications [7, 31, 32].

The effect of chronic alcohol consumption was reported by Daoust et al. [7] with an OR of 1.41. According to Boissoneault et al. [39], chronic alcoholics are predisposed to the phenomenon of hyperalgesia as they have nerve hypersensitivity responsible for a more intense perception of pain. Another hypothesis has been suggested by Apkarian et al. [40] who attributed to alcohol changes in the cerebral cortex or even a “cortical reorganization” responsible for the chronicity of pain. This finding was not confirmed in our study.

Initial management requires a rapid onset of a multimodal analgesia strategy including locoregional analgesic techniques. In fact, epidural analgesia reduces the risk of developing CP. This protective effect is widely studied in the literature [41–44]. It is mainly due to the effects of the epidural on acute pain intensity, which is the most predictable indicator of both prolonged pain and disability with an OR ranging from 1.8 to 1.83 [30–32]. In addition and according to Nejmi et al. [41], early introduction of local anesthetics in the acute phase can stabilize damaged neurons, accelerate neuronal healing, and decrease pain intensity. They block deleterious signals at their origins, reducing the risk of long-term pain.

The effect of sociodemographic characteristics on pain perception in both acute and chronic phases is widely studied in the literature. Rabiou et al. [30] considered that age ≥ 55 years and male gender are two predictors of posttraumatic prolonged chest pain. In the study of Daoust et al. [7], the risk of CP is increased by an age ≥ 65 years (OR = 2.05) and the female gender (OR = 1.49). This finding has also been reported in the context of thoracic and cardiac surgery [45]. Physiopathological mechanisms relied on physiological and psychological components that are difficult to individualize [46]. However, these findings were not confirmed in our study since the relatively heterogeneous aspect of our population with a clear male and juvenile predominance did not allow analysis in sufficiently reliable subgroups.

Other risk factors have been reported in the literature such as the severity of anatomical injuries which explain the high incidence of CP after thoracic surgery. In this context, pain is thought to be secondary to intercostal nerve damage induced by surgical incision, ribs separation, and nerve compression after chest closure. Nevertheless, this mechanical theory does not seem to fully elucidate the pathophysiology of CP since similar proportions have also been observed with the thoracoscopic approach which is less invasive [47].

According to Gordy et al. [33], the flail chest is associated with an increased risk which can nevertheless be reduced by surgical fixation. Two randomized trials evaluating the benefit of surgical fixation of rib fractures concluded that it was associated with shorter length of stay in ICU, shorter duration of mechanical ventilation, earlier rehabilitation, and less risk of CP [48, 49].

Finally, other risk factors should be considered such as the emotional learning circuits of the brain [40], genetic phenomena [50], and properties of the cerebral white matter [51].

The psychosocial impact of CP was assessed in our study using the BPI questionnaire and the PCLS score. The most altered dimensions were general activity, work, sleep, and mood. One- third of patients had probable or very likely PTSD. According to a Singaporean study assessing the quality of life in 23 chest trauma patients, CP was responsible for a 35% reduction in work performance and a psychoemotional impact in 13% of cases [33]. It was responsible for a minimal to moderate disability in 28% and a significant disability in 17% of the cases in the Rabiou et al. study [30].

When evaluating the quality of life of 397 chest trauma patients, Marasco et al. [52] found a return to work rate of 59% at 6 months after trauma. This result has not been improved until 24 months after trauma. The authors report a significant reduction in the quality of life of patients as reflected by the SF-12 quality of life questionnaire and the Glasgow Outcome Scale (GOS) questionnaire.

A 76% disability rate was reported by Fabricant et al. in a series of 110 patients with chronic chest pain. The intensity of pain assessed by the “PPI” score of the Mc Gill “MPQ” questionnaire and the association of several traumatic injuries were predictive of prolonged disability with an OR of 2.2 and 5.9, respectively [31].

In the Shelat et al.'s [32] study, disability defined by functional incapacity had a prevalence of 53%. It was significantly more common in patients with isolated rib fractures. The intensity of the acute pain was a source of functional incapacity in 28% of cases. However, there was no statistically significant relationship between initial injuries and the presence of long-term disability.

In light of these findings, the psychosocial effects of CP following chest trauma can be considered to be frequent and serious. In addition, they are commonly associated with PTSD which is a complication that can occur in anyone who has been exposed to a traumatic event. According to some studies, 69% of the general population is exposed to at least one traumatic event in their lifetime [53] and approximately 5 to 10% experience at least one episode of PTSD [54–57]. This prevalence reaches 9 to 24% among people who have experienced physical trauma [58]. This will depend on several factors widely evaluated in the literature [59].

As reported by Schnurr, CP is often accompanied by PTSD [60]. On the other side and according to Phifer et al. [61], PTSD contributes to the genesis of CP. The incidence of PTSD in patients with previous physical trauma is about 70% and is significantly associated with the severity of acute pain [61], the severity of CP, and the resulting disability [62].

According to Rothschild [63], these associations are explained by interactions between the psychoemotional and somatic areas. Indeed, the vegetative nervous system is activated against any threat of a traumatic situation and it will be “stimulated again with each new sign of trauma, causing somatic flashbacks.”

Asmundson et al. [2] suggest that PTSD and CP are two entities that negatively influence each other and mutually increase the severity of their symptoms via common factors such as fear, avoidance, and tendency to develop catastrophic expectations [64].

According to another literature review which confirmed the frequent association between CP and PTSD, the presence of one or the other of these two disorders is largely sufficient to induce an altered social and relational life [62].

Other experimental studies have tried to explain in part the connection between emotional trauma and the phenomenon of CP. Some have even found a significant correlation between “thinking negatively” and changes in the excitability of the cerebral cortex [65].

## 5. Conclusion

CP following chest trauma is both frequent and severe. Therefore, preventive measures are necessary especially for high-risk patients. They should include the development of risk scores, a better management of acute pain with a proper strategy of multimodal analgesia using in particular locoregional analgesia, a repeated screening strategy based on regular medical monitoring and a multidisciplinary management involving anesthetists, intensivists, algologists, and psychiatrists. However, before any definitive conclusion, these preventive measures must be firstly validated in large scientific studies.

## Figures and Tables

**Table 1:** Sociodemographic and traumatic characteristics of the population.

Variables	Number	Percentage (%)
*Educational status*		
Literacy	4	7.4
Primary	15	27.8
Secondary	24	44.4
Academic	11	20.4

*Tobacco smoking*		
Yes	41	75.9
No	13	24.1

*Alcohol consumption*		
Yes	18	33.3
No	36	66.7

*Sedentary*		
Yes	44	81.5
No	10	18.5

*Comorbidity*		
None	31	57.4
Diabetes	7	13
Hypertension	10	18.5
Others	6	11.1

*ASA status*		
I	31	57.4
II	22	40.7
≥III	1	1.9

*Trauma mechanism*		
Road accidents	29	53.7
Fall	18	33.3
Violence	7	13

*Injuries types*		
Rib fracture	50	92.6
Flail chest	10	18.5
Scapula fracture	7	13
Sternum fracture	5	9.3
Clavicle fracture	3	5.6
Spine fracture	6	11.1
Contusions	28	51.9
Pleural effusion	38	70.3

*Pleural effusion type*		
Pneumothorax	19	50
Hemothorax	15	39.4
Hemopneumothorax	4	10.6

**Table 2:** Chronic pain characteristics.

	Number	Percentage (%)	Mean ± SD
*Intensity of pain at its worst in the last 24 hours*			
0 to 3	9	20.9	4.69 ± 1.59
4 to 6	29	67.4	
7 to 10	5	11.6	
*Intensity of pain at its least in the last 24 hours*			
0 to 3	38	88.4	1.95 ± 1.13
4 to 6	5	11.6	
7 to 10	0	0	

*Intensity of pain on the average*			
0 to 3	24	55.8	
4 to 6	19	44.2	3.18 ± 1.4
7 to 10	0	0	

*Intensity of pain right now*			
0 to 3	29	67.5	
4 to 6	14	32.5	2.79 ± 1.44
7 to 10	0	0	

*Neuropathic pain (DN4* *≥* *3)*			
Yes	39	90.7	
No	4	9.3	

*Neuropathic pain characteristics*			
Tingling	43	100	
Electric shocks	40	93	
Numbness	37	86	
Pins and needles	34	79	
Burning	21	48.8	
Itching	15	34.8	
Painful cold	4	9.3	

SD: standard deviation.

**Table 3:** Associated factors with chronic pain (univariate analysis).

Variables	Chronic pain		*p*
	Yes (*n* = 43)	No (*n* = 11)	
*Pleural effusion*			
Yes (*n* = 38)	34 (89.37%)	4 (10.52%)	0.016
No (*n* = 16)	9 (56.3%)	7 (43.8%)	

*Time to ICU admission*			
≤24 hours (*n* = 49)	41 (83.7%)	8 (16.3%)	0.05
>24 hours (*n* = 5)	2 (40%)	3 (60%)	

*Posttraumatic stress disorders*			
Yes (*n* = 19)	19 (100%)	0	0.017
No (*n* = 35)	24 (68.6%)	11 (31.4%)	

**Table 4:** Psychosocial impact of chronic pain assessed by brief pain inventory (*n* = 43).

Variables	Evaluation	Number	Percentage	Mean ± DS (median)
General activity	[0–3]	28	65.1	3.13 ± 1.74 (3)
	[4–6]	14	32.5	
	[7–10]	1	2.3	
	≥1	43	100	

Mood	[0–3]	29	67.4	2.76 ± 1.84 (3)
	[4–6]	13	30.2	
	[7–10]	1	2.3	
	≥1	39	90.7	

Walking ability	[0–3]	38	88.3	1.1 ± 1.56 (1)
	[4–6]	5	11.6	
	[7–10]	0	0	
	≥1	23	53.5	

Normal work	[0–3]	30	82.5	3.16 ± 2.36 (3)
	[4–6]	10	12.5	
	[7–10]	3	5	
	≥1	40	93	

Sleep	[0–3]	37	86	4.16 ± 2.29 (4)
	[4–6]	3	6.9	
	[7–10]	3	6.9	
	≥1	39	90.7	

Life enjoyment	[0–3]	36	83.7	2.04 ± 1.79 (2)
	[4–6]	6	13.9	
	[7–10]	1	2.3	
	≥1	37	86	

Relations with others	[0–3]	37	86	2.13 ± 1.99 (2)
	[4–6]	3	6.9	
	[7–10]	3	6.9	
	≥1	38	88.4	

## Data Availability

The data used to support this study can be made available from the corresponding author upon request.
